# Nitrosylcobalamin Potentiates the Anti-Neoplastic Effects of Chemotherapeutic Agents via Suppression of Survival Signaling

**DOI:** 10.1371/journal.pone.0001313

**Published:** 2007-12-12

**Authors:** Joseph A. Bauer, Joseph A. Lupica, Heidi Szugye, Bei H. Morrison, Rebecca M. Haney, Rhonda K. Masci, Rebecca M. Lee, Joseph A. DiDonato, Daniel J. Lindner

**Affiliations:** 1 Taussig Cancer Center, Center for Hematology and Oncology Molecular Therapeutics, The Cleveland Clinic Foundation, Cleveland, Ohio, United States of America; 2 Department of Cell Biology, Lerner Research Institute, The Cleveland Clinic Foundation, Cleveland, Ohio, United States of America; 3 Department of Chemistry, Cleveland State University, Cleveland, Ohio, United States of America; 4 Department of Cancer Biology, Lerner Research Institute, The Cleveland Clinic Foundation, Cleveland, Ohio, United States of America; The University of Texas M. D. Anderson Cancer Center, United States of America

## Abstract

**Background:**

Nitrosylcobalamin (NO-Cbl) is a chemotherapeutic pro-drug derived from vitamin B12 that preferentially delivers nitric oxide (NO) to tumor cells, based upon increased receptor expression. NO-Cbl induces Apo2L/TRAIL-mediated apoptosis and inhibits survival signaling in a variety of malignant cell lines. Chemotherapeutic agents often simultaneously induce an apoptotic signal and activation of NF-κB, which has the undesired effect of promoting cell survival. The specific aims of this study were to 1) measure the anti-tumor effects of NO-Cbl alone and in combination with conventional chemotherapeutic agents, and to 2) examine the mechanism of action of NO-Cbl as a single agent and in combination therapy.

**Methodology:**

Using anti-proliferative assays, electrophoretic mobility shift assay (EMSA), immunoblot analysis and kinase assays, we demonstrate an increase in the effectiveness of chemotherapeutic agents in combination with NO-Cbl as a result of suppressed NF-κB activation.

**Results:**

Eighteen chemotherapeutic agents were tested in combination with NO-Cbl, in thirteen malignant cell lines, resulting in a synergistic anti-proliferative effect in 78% of the combinations tested. NO-Cbl pre-treatment resulted in decreased NF-κB DNA binding activity, inhibition of IκB kinase (IKK) enzymatic activity, decreased AKT activation, increased caspase-8 and PARP cleavage, and decreased cellular XIAP protein levels.

**Conclusion:**

The use of NO-Cbl to inhibit survival signaling may enhance drug efficacy by preventing concomitant activation of NF-κB or AKT.

## Introduction

A major obstacle to conventional chemotherapy is the unwanted activation of survival signaling which leads to acquired resistance and decreased therapeutic efficacy. Nuclear factor kappa-B (NF-κB)[1] and Akt [2] are critical mediators of cell survival that are activated following chemotherapy.

NF-κB is a family of heterodimers: NF-κB1 (p50/p105), NF-κB2 (p52/p100), REL, RELA (p65/NF-κB3) in mammals, Dosal, Dif, and Relish in Drosophilia [3]. In its quiescent state, NF-κB is complexed to the inhibitor of κB (IκB) in the cytoplasm. Once phosphorylated , IκB is ubiquitinated and targeted for proteolysis as it remains complexed to NF-κB[4]. Within the proteosome IκB is degraded, while NF-κB is not, allowing NF-κB to translocate to the nucleus where it binds to NF-κB response elements which activate transcription of target genes [5]. NF-κB activation is mediated by kinase cascades that activate IkB kinase (IKK) which is comprised of the subunits IKKα, IKKβ, IKKγ and function to initiate the signal for degradation of IκB[3], [6].

Constitutive activation of NF-κB has been implicated in the development of chemo-resistance in several human carcinoma cell lines[7]–[9]. Low dose doxorubicin can induce drug resistance in cervical carcinoma cells[10]. Human breast cancer specimens contain high levels of NF-κB/RelA indicating constitutive NF-κB activation[11]. High levels of NF-κB and its downstream induced anti-apoptotic genes, bcl-2 and bax correlated with poor response in numerous breast cancer patients[12]. Constitutive NF-κB activity is increased in colorectal cancer[13] and effective treatment is achieved by inhibiting NF-κB[14]. Inhibitor of Kappa B Kinase (IKK), an activator of NF-κB, has been shown to be constitutively active in some prostate carcinoma cell lines[15]. Inhibition of NF-κB increased the efficacy of a variety of chemotherapeutic agents including paclitaxel, etoposide, doxorubicin, cisplatin, 5-FU, irinotecan, CPT-11, and camptothecin [16]–[20] thereby potentiating apoptosis. Similarly, inhibition of AKT can enhance anti-tumor activity of paclitaxel against cervical carcinomas[21].

Akt, a serine/threonine kinase that mediates survival signaling, functions as an oncogene and is implicated in resistance to chemotherapy[22]. AKT1 kinase was found to be elevated in the majority of primary prostate, breast and ovarian carcinomas examined and correlated with high grade and stage of disease[23]. AKT2 was up-regulated in human ovarian[24] and breast carcinomas[25] and was associated with paclitaxel[26] and cisplatin resistance[25]. In addition, decreased activation of AKT via inhibition of phosphoinositide-3-kinase (PI3K) resulted in increased apoptosis of ovarian cancer cells[27]. AKT phosphorylates X-linked inhibitor of apoptosis (XIAP) thereby promoting cell survival[28]. Thus, NF-κB and AKT-mediated survival signaling limit the apoptotic-potential of chemotherapeutic agents suggesting that inhibitors of these pathways can suppress drug resistance and improve therapeutic efficacy.

We have previously demonstrated the anti-tumor activity of nitrosylcobalamin (NO-Cbl) as a single agent and in combination with biological therapies such as IFN-β[29] and Apo2L/TRAIL[30]. NO-Cbl is a vitamin B12 based, nitric oxide donor, that functions as a biological “Trojan Horse” targeting cancer cells via vitamin B12 receptor (Transcobalamin II receptor, TCIIR)[31], [32] mediated uptake[29] similar to clinical studies that target TCIIR in the detection of cancer[33], [34].

We have previously shown that NO-Cbl suppressed Apo2L/TRAIL- and TNF-α- mediated IKK activation with subsequent decreased phosphorylation of IκBα and inhibition of NF-κB DNA binding activity[30]. NO-Cbl sensitized Apo2L/TRAIL-resistant cells to Apo2L/TRAIL-mediated cell death[30]. We have determined that NO-Cbl mediated apoptosis caused in part via activation of the death receptor 4 (DR4) by S-nitrosylation [35]. DR4 mediated apoptosis is tumor specific because Apo2L/TRAIL is overexpressed on the surface of tumors but not on normal tissues such as liver or kidney[36]. Thus, NO-Cbl is a promising candidate to promote apoptosis and minimize toxicity in combination chemotherapy. In the current study, we examined whether NO-Cbl pre-treatment could potentiate the anti-tumor effects of several chemotherapeutic agents.

## Results

### Anti-proliferative effects of NO-Cbl and chemotherapeutic agents *in vitro*

**Figure 1:**
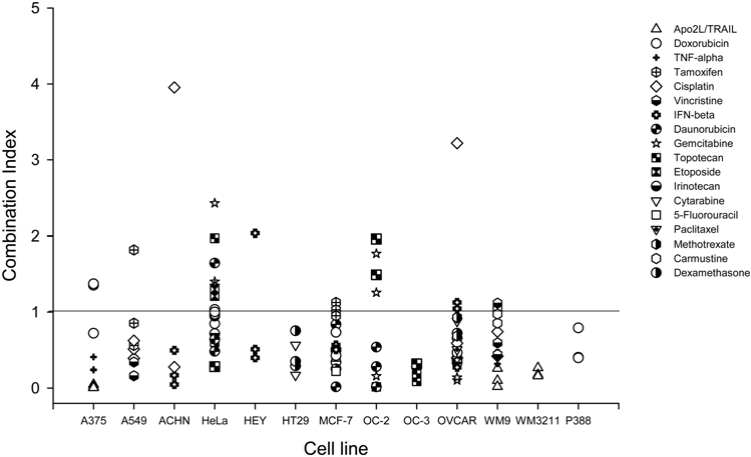
Effects of nitrosylcobalamin (NO-Cbl) and chemotherapeutic agents on the proliferation of A375 (melanoma), A549 (lung), ACHN (renal), HeLa (cervical) HEY (ovarian), HT29 (colon), MCF-7 (breast), OC-2 and OC-3 (platinum and paclitaxel refractory ovarian), OVCAR-3 (ovarian), WM9 and WM3211 (melanoma), and P388 (murine leukemia). Cells were pre-treated with NO-Cbl for 16 h followed by addition of chemotherapeutic agents for five days, and growth was measured by the colorimetric sulforhodamine B assay(37, 75). Data points represent the combination index comparing low, medium, and high combinations of NO-Cbl and each chemotherapeutic agent (mean of eight replicates) to assess synergy. Synergy between NO-Cbl and various chemotherapeutic agents was determined by median effect analysis(38), (combination index >1 indicates antagonism,  = 1 indicates additivity, and <1 indicates synergy). The sequential treatment of NO-Cbl and chemotherapeutic drugs induced synergistic antiproliferative activity in 77.7% of the combinations examined.

To test the hypothesis that NO-Cbl might potentiate the anti-neoplastic effects of various chemotherapeutic agents, we measured antiproliferative effects in thirteen cell lines using 18 chemotherapeutic agents (Figure 1). We used the SRB antiproliferative assay, which is utilized by the National Cancer Institute to evaluate new chemotherapeutic agents[37]. Median effect analysis characterized the interaction between NO-Cbl and the chemotherapeutic agents[38]. Cells were pre-treated with NO-Cbl for 16 h followed by the chemotherapeutic drugs for 80 h. Sequential treatment of tumor cell lines resulted in synergistic antiproliferative activity in 77.78% of the combinations tested, with a mean combination index of 0.44 (95% CI = 0.39 to 0.50). Antagonistic responses were observed in 22.22% of combinations, with an average combination index of 1.73 (95% CI = 1.33 to 2.12).

### NO-Cbl inhibits activation of NF-κB by chemotherapeutic agents

**Figure 2:**
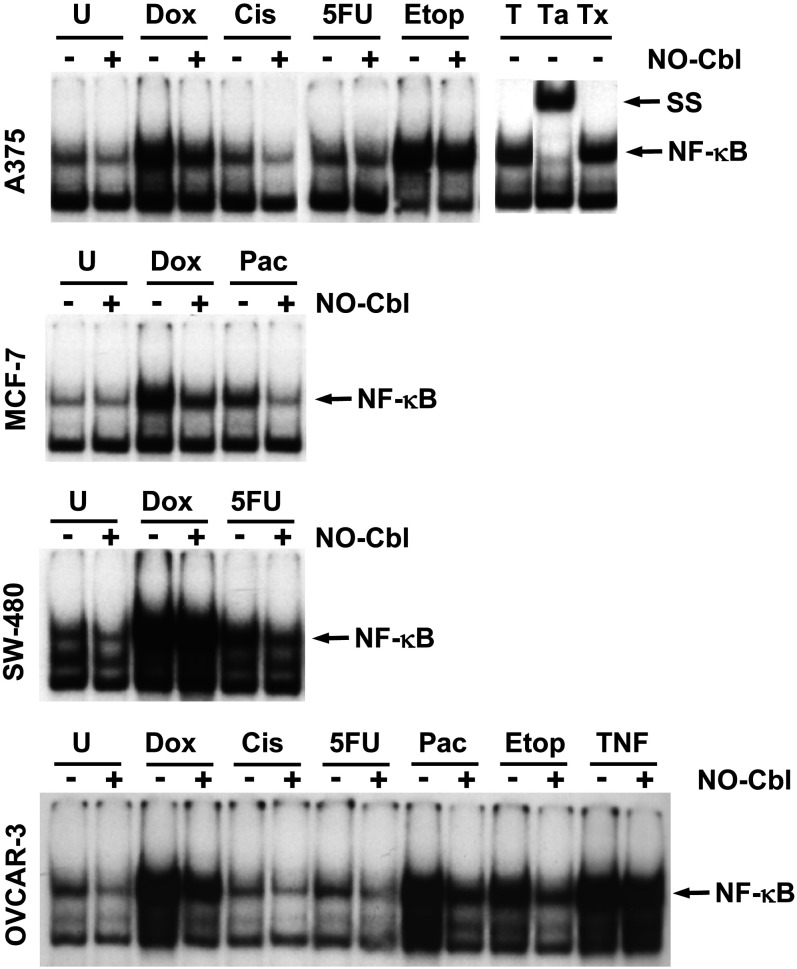
Electrophoretic Mobility Shift Assay (EMSA): NF-κB DNA binding activity. Pretreatment of A375 (melanoma), MCF-7 (breast carcinoma), SW480 (colon carcinoma) and OVCAR-3 (ovarian carcinoma) cells with NO-Cbl (300 µM, 16 h) inhibited the NF-κB DNA binding activity induced by doxorubicin (20 µM, 4 h) or cisplatin (20 µM, 1 h) or 5 flurouracil (5-FU, 100 µM, 5 h) or etoposide (20 µM, 4 h) or paclitaxel (20 µΜ, 5 h). TNF-α (15 min) stimulation served as a positive control of NF-κB activation (T). Incubation of TNF-α treated lysates with anti-NF-κB p65 antibody resulted in supershift (SS) of the NF-κB complex (Ta). An irrelevant antibody was incubated with TNF-α treated lysates which did not result in a supershift (Tx).

EMSA was utilized to examine the effects of NO-Cbl on NF-κB activation by chemotherapeutic agents (Figure 2). We examined four histologically distinct human tumor cell lines [A375 (melanoma), MCF-7 (breast carcinoma), SW480 (colon carcinoma), and OVCAR-3 (ovarian carcinoma)], assaying the effects of multiple chemotherapeutic drugs. TNF-α was used as a positive control for induction of NF-κB DNA binding activity. Specific antibodies to NF-κB-p65 induced a supershift and confirmed the presence of the p65/RelA component of NF-κB.

Doxorubicin treatment resulted in NF-κB activation in all four cell lines. NO-Cbl inhibited NF-κB DNA binding activity in A375 cells by 40.71% (95% CI = 40.52% to 40.87%), MCF-7 cells by 46.99% (95% CI = 46.41% to 47.64%), SW480 cells by 7.52% (95% CI = 5.71% to 9.14%), and OVCAR-3 cells by 40.13% (95% CI = 39.30% to 41.08%).

Cisplatin treatment resulted in low-level NF-κB activation in A375 cells. NO-Cbl inhibited cisplatin-induced activation by 41.54% (95% CI = 40.77% to 42.41%) in A375 cells. Cisplatin did not activate NF-κB in OVCAR-3 cells.

5-FU activated NF-κB in each cell line treated. NO-Cbl inhibited NF-κB activation in A375 cells by 6.89% (95% CI = 6.43% to 7.28%), SW480 cells by 38.14% (95% CI = 34.91% to 40.69%), and OVCAR-3 cells by 18.34% (95% CI = 17.90% to 18.72%).

Etoposide activated NF-κB in A375 and OVCAR-3 cells. Pre-treatment with NO-Cbl inhibited NF-κB DNA binding activity by 29.70% (95% CI = 28.79% to 30.69%) in A375 cells, and 30.37% (95% CI = 30.00% to 30.67%) in OVCAR-3 cells.

Paclitaxel treatment of MCF-7 and OVCAR-3 cells induced NF-κB activation in both cell lines. NO-Cbl inhibited paclitaxel-induced activation by 47.28% (95% CI = 46.23% to 48.43%) and 43.40% (95% CI = 42.48% to 44.47%) respectively, in MCF-7 and OVCAR-3 cells.

### NO-Cbl effects IκB kinase activity

**Figure 3:**
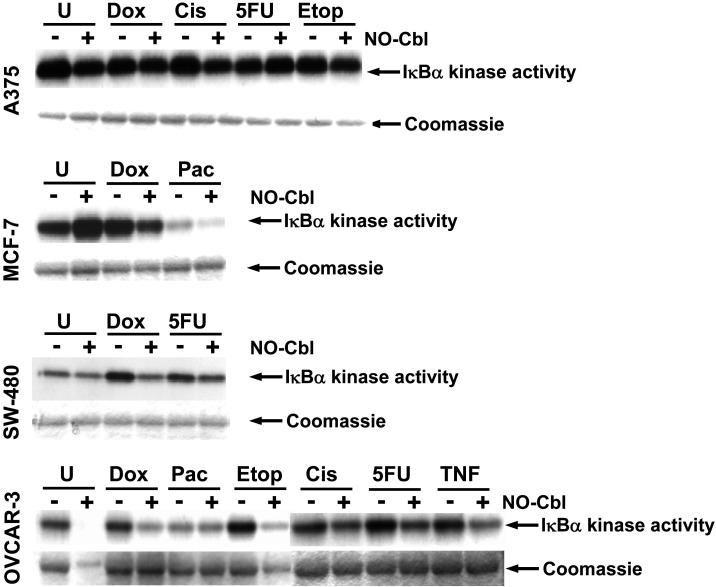
IκB kinase (IKK) activity. IKK activity was assessed using recombinant GST-IκBα(1-54) and γ^32^P-ATP as substrates. The phosphorylated GST fusion protein was detected by autoradiography. IKK activity was determined in cells that were pre-treated with NO-Cbl (300 µM, 16 h) followed by doxorubicin (20 µM, 4 h) or cisplatin (20 µM, 1 h) or 5 flurouracil (5-FU, 100 µM, 5 h) or etoposide (20 µM, 4 h) or paclitaxel (20 µΜ, 5 h). Anti-β-actin antibody was used as an irrelevant antibody control for immunoprecipitation and yielded no signal. After exposure to film, the gel was stained with Coomassie blue to visualize total protein and demonstrated equal loading of the GST-IκBα(1-54) substrate. The same cell extracts were probed for total IKK by immunoblot analysis and demonstrated equal loading of IKK.

IκB kinase (IKK) mediates phosphorylation of IκBα, marking it for eventual polyubiquitination and proteolysis thereby resulting in NF-κB activation[39]. Hence, we examined the effect of NO-Cbl upon IKK activity (Figure 3) using the same lysates used to assess NF-κB DNA binding activity. NO-Cbl treatment reduced basal IKK activity in all treatments, although IKK activity was not noticeably increased in A375 cells following treatment with any of the chemotherapeutic agents.

Doxorubicin activated IKK in MCF-7, SW480 and OVCAR-3 cells. NO-Cbl decreased IKK activity in MCF-7, SW480 and OVCAR-3 cells by 41.70% (95% CI = 39.65% to 44.47%), 45.22% (95% CI = 41.43% to 50.07%) and 50.69% (95% CI = 48.39% to 53.57%), respectively.

Cisplatin treatment resulted in minor activation of IKK only in OVCAR-3 cells. NO-Cbl was effective at inhibiting this activation by 17.06% (95% CI = 15.48% to 19.06%). 5-FU treatment resulted in IKK activation in SW480 and OVCAR-3 cells. NO-Cbl inhibited 5-FU induced IKK activation in SW480 cells by 18.85% (95% CI = 17.20% to 21.05%), and OVCAR-3 cells by 34.12% (95% CI = 31.21% to 37.74%). Paclitaxel did not activate IKK in MCF-7 or OVCAR-3 cells. Treatment of OVCAR-3 cells with etoposide resulted in IKK activation which was inhibited by NO-Cbl by 71% (95% CI = 70.22% to 71.96%).

### Inhibition of AKT activation by NO-Cbl

**Figure 4:**
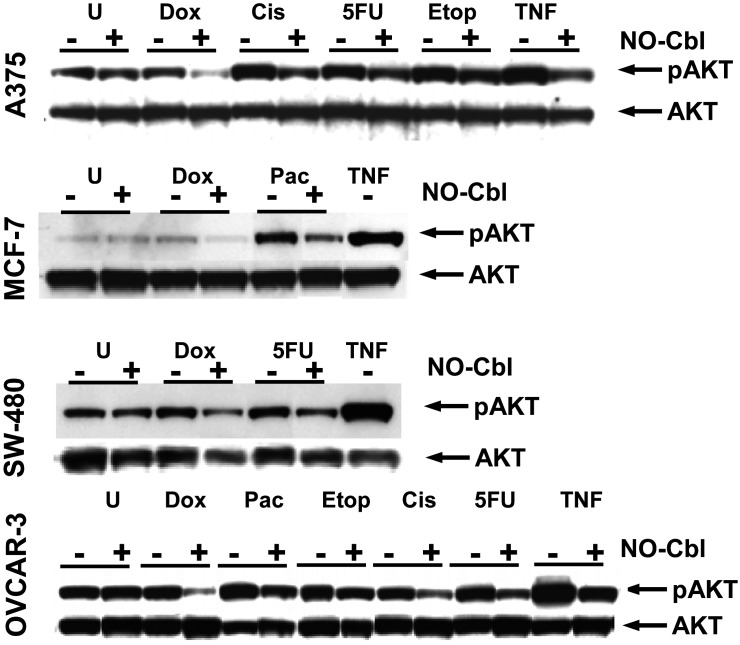
Western blot analysis of phospho-AKT. Cells were pre-treated with NO-Cbl (300 µM, 16 h) followed by doxorubicin (20 µM, 4 h) or cisplatin (20 µM, 1 h) or 5 flurouracil (5-FU, 100 µM, 5 h) or etoposide (20 µM, 4 h) or paclitaxel (20 µΜ, 5 h). Whole cell lysates were probed with anti-phospho-AKT and then re-probed with anti-AKT (unphosphorylated) which served as a loading control.

Next we examined the role of the pro-survival protein AKT and the effects of NO-Cbl on its activation (Figure 4), using the same lysates utilized in the previous two experiments. In A375 cells, phosphorylation of AKT was enhanced following treatment by cisplatin, 5-FU and etoposide. NO-Cbl inhibited AKT activation following cisplatin treatment by 51.24% (95% CI = 50.73% to 51.73%), 5-FU by 42.82% (95% CI = 39.84% to 47.26%), and with etoposide by 46.85% (95% CI = 44.00% to 49.32%).

Doxorubicin increased the phosphorylation of AKT in MCF-7 and SW480 cells which was inhibited by NO-Cbl by 83.77% (95% CI = 81.43% to 86.21%) and 52.60% (95% CI = 49.62% to 55.04%), respectively. Treatment of MCF-7 cells with paclitaxel resulted in increased phosphorylation of AKT which was inhibited by 43.74% (95% CI = 42.01% to 45.00%) following NO-Cbl. Treatment of SW480 cells with 5-FU increased AKT activation which was inhibited by NO-Cbl by 32.50% (95% CI = 31.28% to 33.61%). Although increased phosphorylation of AKT did not occur with any of the chemotherapeutic agents in OVCAR-3 cells, which exhibited a high basal level of phosphorylated AKT, NO-Cbl reduced the high basal AKT phosphorylation levels.

### XIAP expression is decreased with NO-Cbl treatment

**Figure 5:**
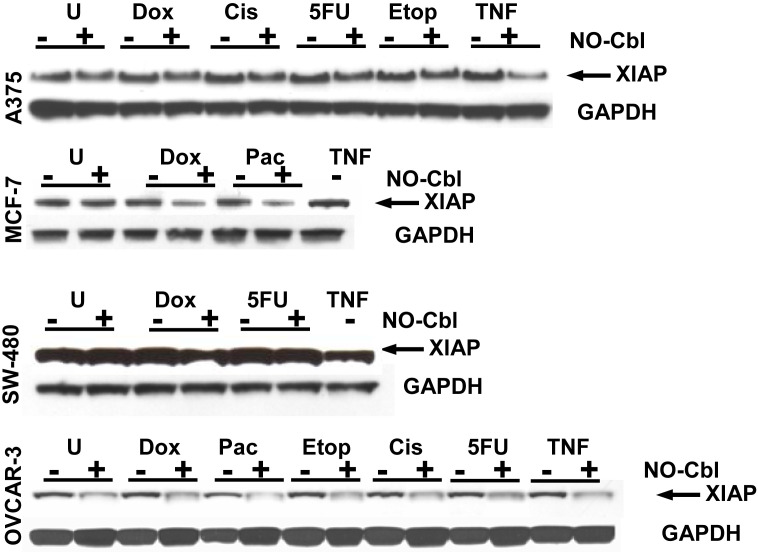
Western blot analysis of XIAP. Cells were pre-treated with NO-Cbl (300 µM, 16 h) followed by doxorubicin (20 µM, 4 h) or cisplatin (20 µM, 1 h) or 5 flurouracil (5-FU, 100 µM, 5 h) or etoposide (20 µM, 4 h) or paclitaxel (20 µΜ, 5 h). XIAP protein levels were determined in whole cell lysates. GAPDH was used as a loading control.

X-linked mammalian inhibitor of apoptosis (XIAP), one of the most potent inhibitors of apoptosis, is a NF-κB-induced gene[40]. Hence we examined effects of NO-Cbl upon downstream gene products induced by NF-κB (Figure 5).

Consistent with AKT phosphorylation in A375 cells, XIAP protein expression was enhanced following doxorubicin, cisplatin, 5-FU, and etoposide treatment. NO-Cbl blunted XIAP expression by 20.12% (95% CI = 14.64% to 23.79%) with doxorubicin, 19.40% (95% CI = 17.06% to 22.00%) with cisplatin, 21.12% (95% CI = 20.89% to 21.31%) with 5-FU, and 18.62% (95% CI = 17.57% to 20.09%) with etoposide.

In addition, doxorubicin increased XIAP expression in MCF-7, SW480, and OVCAR-3 cells; expression was inhibited with NO-Cbl treatment by 44.96% (95% CI = 39.66% to 47.85%), 12.16% (95% CI = 10.57% to 13.79%), and 64.90% (95% CI = 60.46% to 66.85%), respectively. 5-FU treatment increased XIAP expression in SW480 cells which was inhibited with NO-Cbl by 13.66% (95% CI = 11.87% to 15.75%). NO-Cbl treatment inhibited basal XIAP expression when combined with every chemotherapeutic agent in all cell lines examined.

### Effect of NO-Cbl on mediators of apoptosis

**Figure 6:**
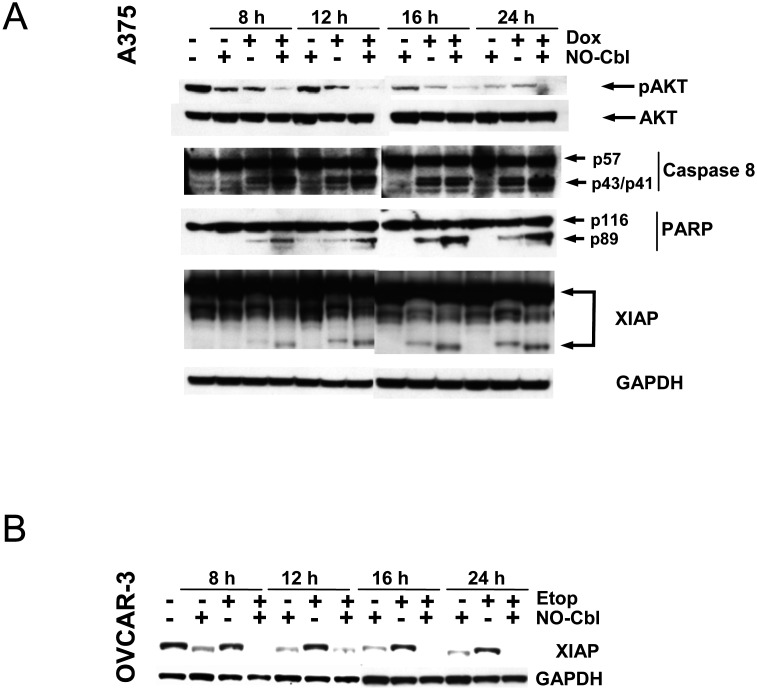
Western blot analysis of mediators of apoptosis. A, Time course analysis of A375 cells pre-treated with NO-Cbl (300 µM, 16 h) followed by doxorubicin (20 µM, 4 h). Phospho-AKT, XIAP, caspase-8 and PARP immunoblots were performed on whole cell lysates. Note that caspase-8 and PARP cleavage were maximal with combination treatment at all time points. Degradation of XIAP was increased following combination treatment at all time points. B, OVCAR-3 cells were pre-treated with NO-Cbl (300 µM, 16 h) followed by etoposide (20 µM, 4 h). XIAP protein levels were determined. GAPDH was used as a loading control.

A375 cells were stimulated with doxorubicin, followed by assessment of AKT phosphorylation, XIAP expression, and caspase-8 and PARP cleavage. (Figure 6a). NO-Cbl inhibited doxorubicin-mediated AKT activation at all time points, resulting in reduction of 85.73% (95% CI = 85.26% to 86.24%) at 8 h, 94.05% (95% CI = 92.44% to 96.02%) at 12 h, 60.94% (95% CI = 58.93% to 63.73%) at 16 h, and completely abrogated the signal at 24 h. In addition, degradation of XIAP was enhanced when NO-Cbl was combined with doxorubicin; this inhibition was 82.94% (95% CI = 81.27% to 84.14%) at 8 h, 49.36% (95% CI = 41.51% to 58.74%) at 12 h, 64.67% (95% CI = 60.68% to 67.69%) at 16 h, and 35.81% (95% CI = 33.93% to 36.54%) at 24 h.

Concordantly, caspase-8 activity was enhanced when NO-Cbl was combined with doxorubicin at 8, 12, 16 and 24 h by 41.39% (95% CI = 27.98% to 47.95%), 36.82% (95% CI = 32.79% to 39.87%), 17.54% (95% CI = 12.22% to 23.98%), and 25.28% (95% CI = 7.88% to 34.92%), respectively. PARP cleavage was increased at all time points following treatment with NO-Cbl and doxorubicin. NO-Cbl enhanced the cleavage of PARP by 82.94% (95% CI = 81.27% to 84.14%) at 8 h, 49.36% (95% CI = 41.51% to 58.74%) at 12 h, 64.67% (95% CI = 60.68% to 67.69%) at 16 h, and by 35.81% (95% CI = 33.93% to 36.54%) at 24 h.

Next, we examined the expression of XIAP in OVCAR-3 cells treated with etoposide and NO-Cbl (Figure 6b). Expression of XIAP protein, normally induced by etoposide, was reduced by NO-Cbl at 8, 12, and 16 h by 92.82% (95% CI = 89.60% to 97.78%), 78.57% (95% CI = 70.12% to 89.26%), and 98.59% (95% CI = 96.71% to 100%), respectively. After 24 h of NO-Cbl treatment, XIAP protein was undetectable.

### Anti-tumor effects of NO-Cbl and chemotherapeutic agents *in vivo*

**Figure 7:**
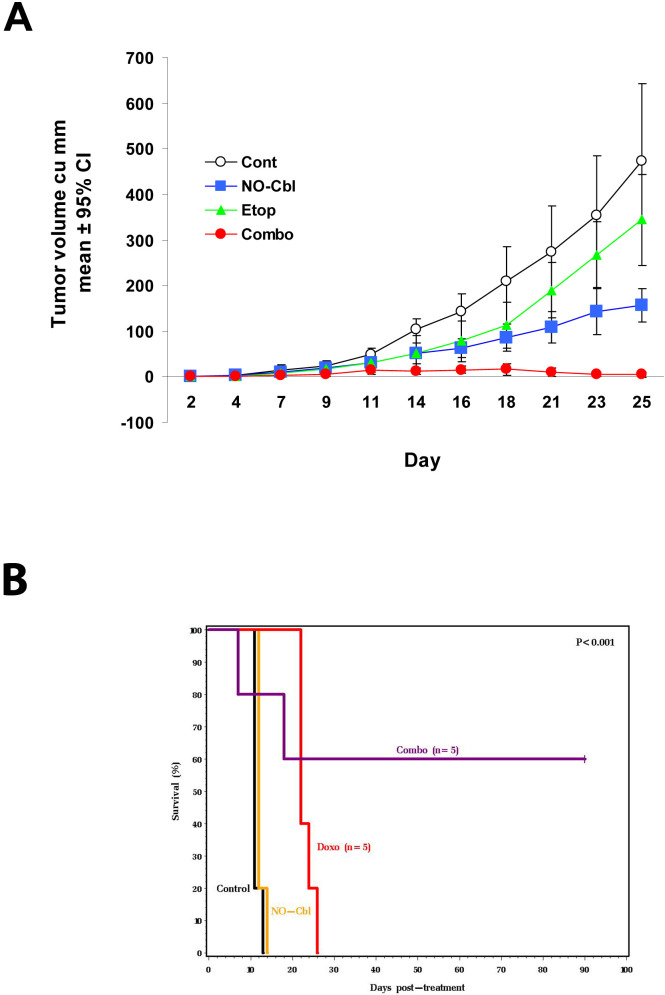
Effects of NO-Cbl and chemotherapeutic agents *in vivo.* A, NCR male athymic nude (nu/nu) mice (*n* = 10 per group) were injected subcutaneously (s.c.) with 2×10^6^ NIH-OVCAR-3 cells. Daily drug treatments of control (PBS), NO-Cbl (150 mg/kg, s.c.), etoposide (2 mg/kg, s.c.), and the combination began on day 2 following inoculation. Tumor volume was measured every other day. Points represent the mean tumor volume±95% CI. B, Kaplan-Meir survival curve. DBA/2 male mice (n = 5) were inoculated intraperitoneally (i.p.) with 10^5^ P388 murine leukemia cells. NO-Cbl was given twice daily (165 mg/kg, i.p.) and doxorubicin (4 mg/kg, i.p.) was administered once weekly, starting on day 2. Treatment in the combination group ceased on day 40 and the animals continued to be monitored for ninety days. Significance comparing the survival of groups was calculated using the logrank test.

*In vitro* combination therapy with NO-Cbl on tumor cells in culture exhibited positive effects in inhibiting cell proliferation and decreasing cell survival signaling (Figs. 1–6). To test drug combinations *in vivo*, subcutaneous NIH-OVCAR-3 xenografts were established in athymic nude mice. Daily drug treatments began on day 2 after cell inoculation, at which time tumors were both visible and palpable (Figure 7a). After 25 days, the tumors from mice treated with NO-Cbl were reduced in volume by 66.94% (95% CI = 60.90% to 69.81%; *P* = 0.00068) compared to controls. Tumors from mice treated with etoposide were smaller by 27.30% (95% CI = 19.70% to 30.90%; *P* = 0.14099) compared to control tumors. Mice receiving the combination of NO-Cbl and etoposide displayed tumors that were inhibited by 99.01% (95% CI = 98.48% to 100.12%; *P* = .000012) compared to controls. In two mice, tumors disappeared completely and did not recur 60 days after treatment cessation.

In a syngeneic P388 murine leukemia model, cells were inoculated into the peritoneal cavities of DBA/2 mice. Daily drug treatments began on day 2 following inoculation (Figure 7b). Untreated animals died by day 14. NO-Cbl treated animals survived until day 21. Doxorubicin treated animals survived until day 33. Combination drug treatment was discontinued on day 40 and resulted in 60% survival, monitored through day 90. Logrank analysis determined P<0.001 comparing Kaplan Meier survival curves.

## Discussion

Nitric oxide (NO) is a ubiquitous multi-faceted signaling molecule critical to many physiological and pathological processes[41]. A comprehensive review by Mocellin et al. details the use of NO donors to induce apoptosis[42]. The connection between NO donors and apoptosis often seems paradoxic and depends upon cell type, site of delivery, and NO concentration. However, it is widely accepted that high levels of NO (>1 µM) can activate the extrinsic pathway of programmed cell death via protein nitrosylation[35], [43] and can also activate the intrinsic pathway via oxidative stress and cytochrome c release[44]. Most conventional NO donors, especially those with short half-lives such as GTN, SNP and SNAP, induce significant toxicity to normal cells due to rapid NO release in biological fluids[45], [46], a drawback to their use.

A major advantage of the pro-drug NO-Cbl is its tumor-specific accumulation due to higher transcobalamin receptor (TCII-R) expression in tumor cells compared to normal tissues[29]. Cobalamin (Cbl) is avidly taken up by tumor cells relative to most normal tissues[34], [47], [48] Unlike other donors, NO-Cbl preferentially releases NO inside the cell, and therefore minimizes systemic toxicity due to high plasma NO concentration. By taking advantage of the “Trojan Horse” properties of NO-Cbl, side effects such as vasodilation can be minimized. We have not observed hypotension, bone marrow suppression or abnormal liver or kidney function in our rodent or canine studies following acute or chronic NO-Cbl administration (data not shown).

NO inhibits survival signaling via inhibition of NF-κB [49], [50] and AKT [51], both well characterized mediators of cell survival. A recent review by Nakanishi and Toi implicates NF-κB activation as a major contributor to lowering the apoptotic efficacy of chemotherapeutic agents and increasing drug resistance[52].

The activation of NF-κB is a highly complex process. There are cell type-specific variations in NF-κB activation that differ between cell lines as well as activation differences among known NF-κB-activators. Taxol treatment of pancreatic cancer cell lines induces formation of several different NF-κB/IκB complexes, suggesting the presence of multiple up-stream activators of NF-κB[53]. UV-induced DNA damage in HeLa cells is characterized by IKK-independent activation of NF-κB at early time points (30 min) and IKK-dependent activation at later times (15–20 h)[54]. Doxorubicin initiates phosphoinositide-3-kinase (PI3K)-mediated degradation of IκB that is independent of the IKK signalosome[55]. Conversely, both PI3K and AKT can mediate NF-κB activation via phosphorylation of threonine 23 of IKKα, leading to NF-κB activation[56]. In comparison, AKT required IKK in order to activate NF-κB[57], specifically IKKβ[58]. In addition, AKT potentiates the effects of gemcitabine and paclitaxel in pancreatic cancer possibly via NF-κB activation[59]. Similarly, AKT can transiently bind and activate IKK following stimulation with platelet-derived growth factor (PDGF)[60]. In melanoma, AKT may activate NF-κB in an IKK-independent fashion[61] possibly mediated by MAPK[62]. Undoubtedly, the complex nature of NF-κB activation can be as paradoxic as the cellular actions of NO.

Our data demonstrates consistent inhibition of NF-κB by NO-Cbl, irrespective of the survival pathways induced by a variety of chemotherapeutic agents. We demonstrate increased NF-κB DNA binding induced by almost every chemotherapeutic agent across all cell lines. Doxorubicin, etoposide, and paclitaxel induced NF-κB activation in all cell lines examined (Figure 2). Interestingly, A375 cells had greater constitutive IKK activation compared to MCF-7, SW480, and OVCAR-3 cells, and IKK activity in A375 cells was not noticeably enhanced by any chemotherapeutic agent (Figure 3). Yet, treatment of A375 cells with NO-Cbl reduced basal IKK activity. The absence of increased IKK activity in A375 cells following chemotherapeutic drug treatment may be attributed to: 1) differences in the kinetics of activation of IKK vs. NF-κB, and 2) preferential phosphorylation of the GST-IκBα(1-54) substrate by IKK-β rather than IKK-α [63]. These factors may result in understated IKK-α contributions to activation using the current kinase assay. Admittedly, IKK-α may play a more dominant role in NF-κB activation as compared to IKK-β mediated NF-κB activation in A375 cells, but this topic is ancillary to the conclusion of this study. The key observation is that where NF-κB is activated, NO-Cbl inhibits the activation and furthermore, in activation pathways that clearly utilize IKK to trigger NF-κB activation, NO-Cbl treatment inhibits IKK activity.

Conversely, AKT was activated by cisplatin, 5-FU, and etoposide in A375 cells. NO-Cbl markedly reduced AKT phosphorylation following all treatments (Figure 4). Thus, AKT may be involved in NF-κB activation, independent of IKK, as previously reported [61]. This may also explain activation of NF-κB in MCF-7 cells following treatment by paclitaxel, accompanied by AKT activation in the absence of IKK activation (Figure 4). Alternatively, AKT and IKK may act in concert to activate NF-κB [60]. In OVCAR-3 cells, AKT was not activated by any chemotherapeutic agent whereas IKK activation was induced by doxorubicin, etoposide, cisplatin, and 5-FU, suggesting IKK-dependent activation in these cells (Figure 4). Our results with paclitaxel are in agreement with results obtained by others in ovarian cancer cells using paclitaxel [64]. Clearly, NF-κB signaling is a complex process involving several pathways that provide diverse control points resulting in a balance between apoptosis and survival.

NO-Cbl inhibited expression of a NF-κB-induced survival factor, namely X-linked inhibitor of apoptosis (XIAP) (Figure 5), consistent with other studies in which S-nitrosoglutathione (GSNO) inhibited XIAP expression[65]. Our data suggest that the inhibitory effects of NO-Cbl on XIAP are post-translational as well as transcriptional (Figure 5). The increase in caspase-8 activation, PARP cleavage and XIAP degradation are consistent with the synergistic anti-proliferative effects of NO-Cbl (Figure 6) and provide further evidence as to the chemopotentiating effects of NO-Cbl. Elimination of OVCAR-3 and P388 tumors *in vivo* (Figure 7) is consistent with the antiproliferative synergy observed *in vitro*.

We are continuing to study the mechanism by which NO-Cbl inhibits NF-κB activation. This likely involves S-nitrosylation [66]. Prostaglandins (PGA_1 _and 15dPGJ_2_) inhibit IKK by covalently modifying a critical cysteine residue (C179) within the activation loop [67]. S-nitrosylation of IKK-β leads to its inactivation[68]. In addition, the NO donor SNAP has been shown to inhibit IKK-β[69] as well as suppress AKT activation[51]. Also, inactivation of AKT can result from S-nitrosylation and may be involved in insulin resistance[70]. Alternatively or in addition, both IKK-γ and XIAP contain zinc fingers which are known targets for nitrosylation [71]. Others have shown that NO can inhibit NF-κB by direct S-nitrosylation of the DNA binding subunits [49], [50], [72] or tyrosine nitration[73].

Therefore, NO-Cbl likely nitrosylates redox-sensitive residues in its target proteins, causing inhibition of NF-κB-mediated survival signaling and this appears to also be the case with AKT-mediated survival signaling. We have shown that NO-Cbl maximizes the anti-tumor effectiveness of primary chemotherapeutic agents. The use of NO-Cbl to inhibit survival signaling may prevent drug resistance, a common occurrence in multiple cycle chemotherapy, and may improve response rates and enhance drug efficacy.

## Materials and Methods

### Synthesis of Nitrosylcobalamin

Nitrosylcobalamin was synthesized as previously described[74]. Hydroxocobalamin (vitamin B12a) acetate (Hebei Huarong Pharmaceutical Co, Hebei Province, China) was dissolved in dichloromethane (OmniSolv, EMD Chemicals, Gibbstown, NJ) and exposed to CP grade NO gas (Praxair, Wickliff, OH) at 75 psi. The reaction proceeds in a closed system within a high-pressure stainless steel reactor (Parr Instrument Co, Moline, IL). The system was purged daily and evacuated prior to NO exposure. The NO gas was scrubbed prior to entering the system using a stainless steel cylinder (Midwest Process Controls, Bay Village, OH) containing NaOH pellets. The solid NO-Cbl product was collected following rotary evaporation of the solvent and stored under argon at −80°C prior to use.

### Cell Culture and Cytokine treatments

Human tumor cell lines (ATCC, Manassas, VA) were grown in RPMI 1640 and Dulbecco's modified Eagle medium as appropriate (DMEM, Cellgro, Mediatech Herndon, VA) supplemented with 10% fetal bovine serum (FBS, Mediatech) and 1% Antibiotic-Antimycotic (GIBCO BRL, Invitrogen, Carlsbad, CA) according to ATCC recommendations. Cells were maintained in 5% CO_2_ at 37°C in a humidified incubator. Cells were confirmed as mycoplasma free using a commercially available kit (MycoAlert, Cambrex Corporation, East Rutherford, NJ).

### Sulforhodamine B Cell Growth Assay

Cells were harvested with 0.5% trypsin/0.53 mM EDTA, washed with PBS and resuspended in media containing 10% FBS. Cells were plated in 96-well plates in 0.2 mL aliquots containing 2,000 cells. Cells were allowed to adhere to the plate for 4 h and then NO-Cbl was added in different dilutions to the assay plate. Replicates of eight were performed for each treatment. After 16 h, chemotherapeutic agents were added at different concentrations. Growth was monitored by the sulforhodamine B (SRB, Sigma Chemical, St. Louis, MO) colorimetric assay[37], [75]. After 80 h growth, the medium was removed, and cells were fixed with 10% trichloroacetic acid and stained with SRB. Bound dye was eluted from cells with 10 mM TRIS (pH 10.5) and absorbance was measured at 570 nm using a Lab Systems Multiskan RC 96-well plate reader (Lab Systems Multiscan RC, Thermo Lab Systems, Franklin, MA). To quantify growth of the cells, experimental absorbance values (A_exp_) were compared with initial absorbance readings representing the starting cell numbers (A_ini_). To determine the starting cell number, an additional 96-well plate was seeded with cells and fixed at the beginning of the experiment. The absorbances derived from the initial plate and from the untreated cells at the end of the growth period (A_fin_) were defined as 0% and 100% growth, respectively. The percentage control growth (100%×[A_exp_–A_ini_]/[A_fin_–A_ini_]) was expressed as a percentage of untreated controls.

### *In vivo* experiments

Five week-old NCR male athymic nude homozygous *(nu/nu*) mice (Taconic, Germantown, NY) were inoculated with NIH-OVCAR-3 tumors. There were four experimental groups (untreated, single agents, and the combination), n = 10. Tumor cells (2×10^6^) were inoculated into flanks in the mid-axillary line. NO-Cbl was given once daily (150 mg/kg, s.c.) and etoposide (2 mg/kg, s.c.) was administered once weekly, starting on day 2. Tumor volume was measured three times a week using the formula for a prolate spheroid: (4/3) πab^2^ where 2a = major axis, 2b = minor axis. Formalin-fixed sections were processed by the Cleveland Clinic Histology Core. Sections were stained with hematoxylin and eosin and evaluated for pathologic changes in a blinded fashion. For syngeneic studies, five week-old DBA/2 male mice (Taconic) were inoculated with P388 murine leukemia cells. There were four experimental groups (untreated, single agents, and the combination), n = 10. Tumor cells (10^5^) were inoculated i.p.. NO-Cbl was given twice daily (165 mg/kg, i.p.) and doxorubicin (4 mg/kg, i.p.) was administered once weekly, starting on day 2. Animals were monitored for ninety days. The Institutional Animal Care and Use Committee at the Cleveland Clinic Foundation approved all procedures for animal experimentation.

### Gel Electrophoresis and Immunoblot analyses

Cells were pre-treated with NO-Cbl (300 µM) for 16 h followed by doxorubicin (20 µM, 4 h), cisplatin (20 µM, 1 h), 5 flurouracil (5-FU, 100 µM, 5 h), etoposide (20 µM, 4 h), or paclitaxel (20 µM, 5 h). As a positive control for NF-κB induction, cells were treated with TNF-α (20 ng/mL, 15 min). Whole cell lysates were prepared in 1× high salt lysis buffer (20 mM HEPES, 400 mM NaCl, 25 mM β-glycerol phosphate, 25 mM NaF, 10 mM, para-nitrophenyl phosphate (PNPP), 10% glycerol, 0.5 mM NaOrthovanadate, 1mM PMSF, 0.5% NP40 and 1× protease inhibitor cocktail set I (Calbiochem) and 1× phosphatase inhibitor cocktail set II (Calbiochem) for subsequent immunoblotting studies. Total cell protein (80 µg per sample) was resolved by electrophoresis on a 10% Bis-TRIS gel (Criterion, Bio-Rad, Hercules, CA) resolved at 160 V for 1 hour and transferred to a PVDF membrane (Immobilon-P, Millipore, Bedford, MA) with the use of a wet transfer apparatus (Bio-Rad) for 30 min at 100 V . The membranes were incubated in blocking buffer (Starting Block T20, Pierce, Rockford, IL) for 1 h at room temperature and then incubated overnight with primary antibody [anti-phospho-AKT (Ser473) 1∶1000, Cell Signaling, Boston, MA; anti-AKT 1∶1000, Cell Signaling; anti-XIAP, 1∶250-2000, BD Biosciences, San Jose CA; anti-Caspase-8 (1C12) 1∶1000 Cell Signaling; anti-PARP 1∶1000, Cell Signaling] diluted in Starting Block. After washing in 1× TBST (USB Corp, Cleveland, OH) membranes were incubated with horseradish peroxidase-conjugated goat secondary antibodies (Biorad) diluted in Starting Block for 1 h at room temperature. The protein bands were visualized by enhanced chemiluminescence (SuperSignal West Pico chemiluminescent substrate, Pierce). Antibodies against glyceraldehyde-3-phosphate dehydrogenase (Trevigen, Gaithersburg, MD) were used to ensure equal loading.

### Electrophoretic mobility shift assay (EMSA)

Cells were pre-treated with NO-Cbl (300 µM) for 16 h followed by doxorubicin (20 µM, 4 h) or cisplatin (20 µM, 1 h) or 5 flurouracil (5-FU, 100 µM, 5 h) or etoposide (20 µM, 4 h) or paclitaxel (20 µΜ, 5 h). As a positive control for NF-κB induction, cells were treated with TNF-α (20 ng/mL, 15 min). Plates were washed three times with ice-cold 1× DPBS (Cellgro, Mediatech). Cells were scraped from plates and resuspended in 1× high salt lysis buffer as above. The lysates were transferred to pre-chilled microcentrifuge tubes and incubated at 4°C using a vortex for 30 min followed by centrifugation at 20,000g for 15 min. Samples were snap frozen with liquid nitrogen and stored at −70°C until needed. Supernatants were transferred to fresh tubes and protein concentrations were assessed using the Bradford method (Bio-RAD protein assay, BioRad, Hercules, CA). We used a commercially available consensus binding site for NFκB/c-Rel homodimeric and heterodimeric complexes as a probe to assess DNA binding activity. The NF-κB consensus binding sequence (5′AGTTGA**GGCGACTTTCCC**AGGC 3′) (sc-2505: Santa Cruz Biotechnology, Santa Cruz, CA) was end-labeled with γ^32^P-dATP (3000 Ci/mol, Perkin Elmer, Boston MA), using T4 polynucleotide kinase (Roche, Indianapolis, IN). DNA binding reactions were performed in 28 µL reaction volume for 30 min on ice containing 20 µg protein, 100 mM HEPES, 3.0 mM EDTA, 50% glycerol, 3 mM DTT, 25 mM MgCl_2_, 20 mM TRIS, pH 7.90, 5 ug Poly [d(I-C)] and labeled probe. Complexes were separated from the free probe on 6% non-denaturing polyacrylamide gels in 0.5× TBE buffer at 195 V for 2 h. Gels were dried and exposed to film. To verify that the band shifts were comprised of NF-κB, lysates from cells that were stimulated by TNF-α (20 ng/mL, 15 min) were incubated with anti-NF-κB p65 antibody (100 ng/mL, Zymed, S. San Francisco, CA).

### IκB kinase (IKK) assay

Whole cell extracts (200 µg) treated as above were supplemented with 150 µL of Buffer A (20 mM Hepes, pH 7.9, 20 mM beta-glycerophosphate, 10 mM NaF, 0.1 mM orthovanadate, 5 mM para-nitrophenyl phosphate (PNPP), 10 mM 2-mercaptoethanol (BME), 0.5 mM PMSF, and protease cocktail), 2 µL normal rabbit serum (NRS), and mixed by rotation at 4°C for 1 h as previously described[76]. A 50% slurry of Protein G Sepharose (80 µL) (Amersham-Pharmacia, Piscataway, NJ) prepared in Buffer A (without BME or PMSF) was added and mixed by rotation at 4°C for 1 h. Protein G Sepharose was removed by centrifugation at 800g for 1 min and discarded. Anti-IKKα monoclonal antibody (0.5 µg, BD-Pharmingen, San Diego, CA), or anti-HA epitope antibody (Covance, Berkeley, CA) was added to the supernatant and mixed by rotation at 4°C for 2 h. A 50% slurry of Protein G Sepharose (60 µL) prepared in Buffer C (Buffer A plus 50 mM NaCl and 10 mM MgCl_2,_ without BME and PMSF) was added and mixed by rotation in the cold for 30 min. Protein G immunopellets were collected by centrifugation at 800 g for 30 sec, washed 3 times with Buffer B (Buffer A plus 250 mM NaCl), and once with Buffer C (Buffer A plus 50 mM NaCl and 10 mM MgCl_2_). Immunopellets were re-suspended in 30 µL kinase buffer with 0.1 mM orthovanadate, 50 µM unlabeled ATP, 5 µCi γ^32^P-ATP, 2 mM DTT, and 2 µg of recombinant GST-IκBα1-54[39] and incubated at 30°C for 30 min. Reactions were stopped by the addition of 15 µL 4× SDS-PAGE loading buffer (200 mM Tris-HCl, pH 6.8, 8% SDS, 40% glycerol, 0.2% 2-mercaptoethanol), heated at 95°C for 10 min, and resolved by SDS-PAGE on a 12% acrylamide gel by standard procedures. Gels were rinsed, stained with Bio-Safe Coomassie (BioRad, Hercules, CA) to visualize protein bands, rinsed, photographed then dried and exposed to Kodak BioMax MR film (Eastman Kodak Co., Rochester, NY) to detect substrate phosphorylation. IKK activation was quantified by PhosphorImage analysis on a Storm-840 imager using Image Quant v 5.2 software (Molecular Dynamics, Amersham Biosciences, Piscataway, NJ).

### Statistical Analysis

Median effect analysis was used to characterize the interaction between NO-Cbl and various chemotherapeutic agents[38]. A combination index >1 indicates antagonism,  = 1 indicates additivity, and <1 indicates synergy. Unless otherwise stated, results are expressed as means with 95% confidence intervals (95% CI). Differences in mean tumor volume between groups were compared using the unpaired two-tailed Student's *t* test, using a pooled estimator of variance, to determine statistical significance using Sigma Plot 10.0 (SPSS, Chicago, IL). The logrank test was used to calculate the significance of the Kaplan Meir survival curve. The experimental design utilized the same set of cell lysates for Figures 2-5, and the data was expressed as means (n = 3).
